# Photon transport through the entire adult human head

**DOI:** 10.1117/1.NPh.12.2.025014

**Published:** 2025-05-28

**Authors:** Jack Radford, Vytautas Gradauskas, Kevin J. Mitchell, Samuel Nerenberg, Ilya Starshynov, Daniele Faccio

**Affiliations:** University of Glasgow, School of Physics and Astronomy, Glasgow, United Kingdom

**Keywords:** biophotonics, diffuse optical tomography, transmittance, photon counting, functional near-infrared spectroscopy

## Abstract

**Significance:**

The highly scattering nature of near-infrared light in human tissue makes it challenging to collect photons using source-detector separations larger than several centimeters. The limits of detectability of light transmitted through the head remain unknown. Detecting photons in the extreme case through an entire adult head explores the limits of photon transport in the brain.

**Aim:**

We explore the physical limits of photon transport in the head in the extreme case wherein the source and detector are diametrically opposite.

**Approach:**

Simulations uncover possible migration pathways of photons from source to detector. We compare simulations with time-resolved photon counting experiments that measure pulsed light transmitted through the head.

**Results:**

We observe good agreement between the peak delay time and width of the time-correlated histograms in experiments and simulations. Analysis of the photon migration pathways indicates sensitivity to regions of the brain well beyond accepted limits. Source repositioning can isolate sensitivity to targeted regions of the brain, including under the cerebrum.

**Conclusions:**

We overcome attenuation of $∼10^{18}$ and detect photons transmitted through an entire adult human head for a subject with fair skin and no hair. Photons measured in this regime explore regions of the brain currently inaccessible with noninvasive optical brain imaging.

## 1 Introduction

Optical modalities for noninvasive imaging of the human brain hold promise to fill the technology gap between cheap and portable devices such as electroencephalography (EEG) and expensive high-resolution instruments such as functional magnetic resonance imaging (fMRI), with devices that have high sample rates, high spatial resolution, and are relatively inexpensive. A major bottleneck for the widespread adoption of optical brain reading devices in clinics and neuroscience studies is the low number of photons emerging from deep layers of the brain that restrict the sensitivity of these methods to a maximum of 4 cm below the scalp,^1^^–^^3^ corresponding to the outermost layer of the cerebral cortex (gyri). However, imaging paradigms have been demonstrated using highly scattered transmitted photons in thick diffusive materials,^4^^,^^5^ and it has been theoretically shown that photons experiencing similar scattering length scales as the diameter of the human head can carry imaging information.^6^ Therefore, extending current optical methods to extract information about deep brain regions that are currently inaccessible, e.g., cortical folds (sulci), midbrain, and deep regions of the cerebellum, may be feasible with careful consideration of sources and detectors and incorporating time-resolved photon counting information so long as photons are detected through the extreme number of attenuation lengths.

The most common optical approach to infer brain activity measures differential changes in the absorption of near-infrared light to determine blood oxygenation levels. One of the pioneering works to demonstrate this technique was reported by Jobsis^7^ and later led to the field of functional near-infrared spectroscopy (fNIRS). Current fNIRS devices have faster sample rates than fMRI and better spatial resolution than electroencephalograms (EEG).^8^^,^^9^ Diffuse optical tomography (DOT) extends fNIRS to include depth information, which can be used to selectively resolve changes in blood chromophores in superficial and cortical layers.^10^^–^^13^

Unfortunately, there remain outstanding challenges associated with the use of optical and near-infrared wavelengths in the adult human head, the most prominent of which is the shallow depth of sensitivity under the scalp that restricts fNIRS to monitor only the outermost region of the cortex. This limitation is due to the highly scattering nature of human tissue, which causes an exponential attenuation of light for increasing penetration depth. Liu et al.^14^ attempted to measure deep brain activity with fNIRS using a model that maps coregistered fNIRS to fMRI data and can be used to infer activity in deep regions (sulci) from surface-level (gyri) optical measurements. However, a direct measurement of this information is more accurate than relying on correlations between fNIRS and fMRI, which monitor brain activity by fundamentally different physical mechanisms.

In the claims of the early work by Jobsis,^7^ a signal is presented highlighting an increase in transmission of near-IR light diametrically from temple to temple in an adult human head due to a decrease in cerebral blood volume during hyperventilation. Unfortunately, the results were incomplete because the experiment was stopped before the signal could return to baseline. Since this work, the only studies to detect light diametrically (i.e., across the widest point of the skull) through the head involve neonatal or infant subjects that have more transparent skulls and significantly smaller diameter heads compared with adults. Studies in the infant brain have shown that fNIRS allows for 3D tomographic reconstructions of brain activity and cerebral hemodynamics of the entire head.^12^^,^^15^^,^^16^ However, using transmitted light to produce tomographic maps in this way for the adult head has yet to be demonstrated. Indeed, some suggest that the detection of light diametrically through an adult human head is “impossible.”^17^ A back-of-the-envelope estimate very simply considers only the white matter region (L=10  cm thick, $\mu_{a}=0.92\;{\mathrm{cm}}^{-1}$, $\mu_{s}^{\prime }=49.4\;{\mathrm{cm}}^{-1}$^18^), and an exponential Beer-Lambert decay $∼\exp(-\sqrt{3\mu_{a}(\mu_{a}+\mu_{s}^{\prime }})L$^19^ would indicate a remarkably high attenuation of $10^{53}$, thus supporting the apparent impossibility of detecting photons that have propagated diametrically through the brain.

In this work, we explore the details of photon transport through the head and present numerical and experimental evidence that photons can actually be transmitted diametrically through the entire adult human head, albeit along specific trajectories. We find a wide variety of light propagation pathways that are largely determined by the presence of and guided by cerebrospinal fluid. Different source positions on the head can then selectively isolate and probe deep regions of the brain. This suggests that optical techniques could be used to monitor activity in the sulci, midbrain, and deep regions of the cerebellum, which are currently inaccessible with fNIRS.^20^ Healthcare applications such as sensing or imaging strokes and tumors at point-of-care may also benefit from systems inspired by the results of this work. These findings uncover the potential to extend noninvasive light based on brain imaging technologies to the tomography of critical biomarkers deep in the adult human head.

## 2 Materials and Methods

### 2.1 Numerical Modelling

**Fig. 1:**
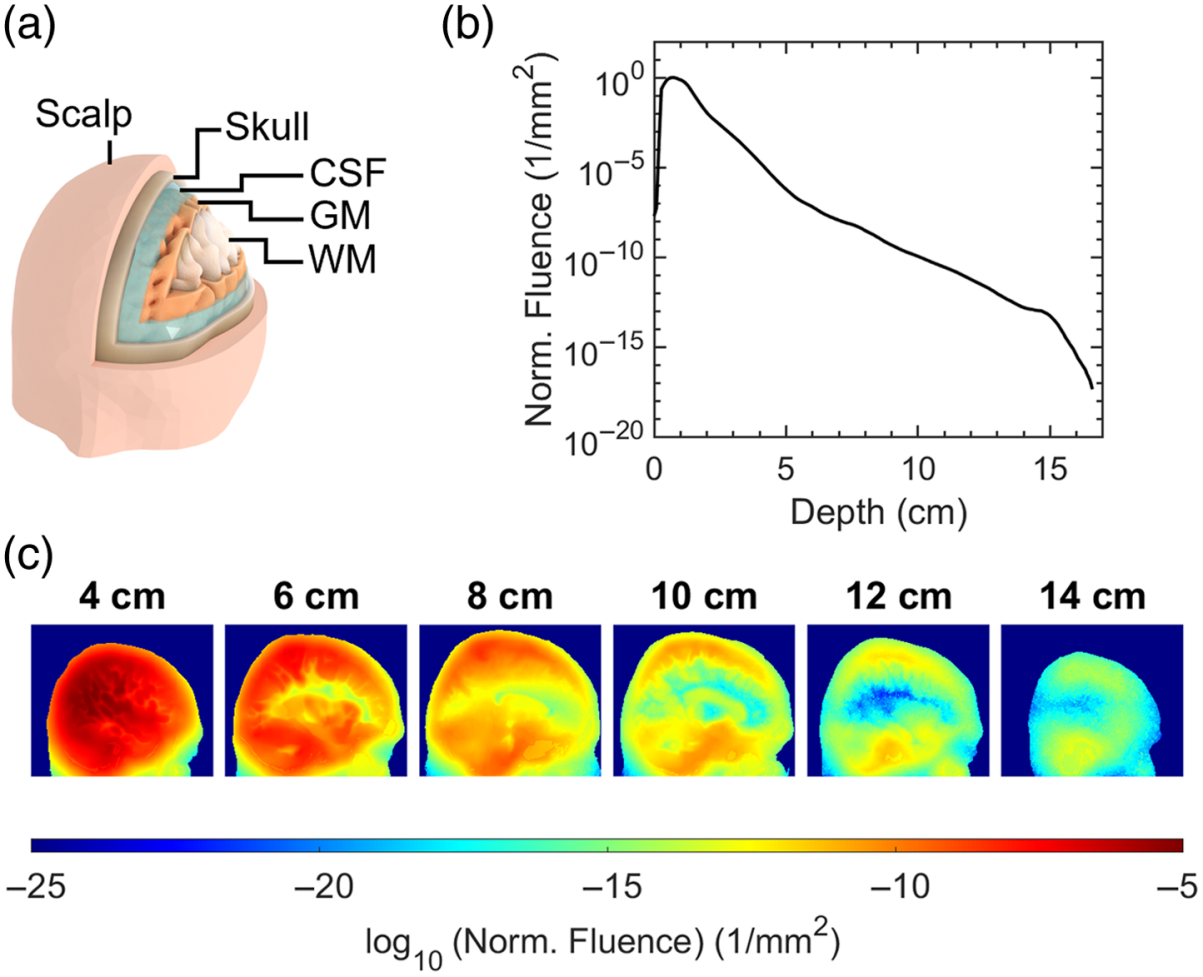
Numerical simulations. (a) A smoothed render of the head volume mesh used in simulations highlighting the layers considered in this work. (b) The normalized fluence ($1/{\mathrm{mm}}^{2}$) inside the head for increasing the distance from the source. (c) The distribution of fluence ($1/{\mathrm{mm}}^{2}$) at various sagittal slices plotted in logarithmic scale—light preferentially persists in regions of low absorption and scattering.

Monte Carlo ray-tracing simulations were performed using Monte Carlo eXtreme (MCX) to estimate the attenuation and distribution of light through the head. An open-source six-layer head volume mesh [Fig. 1(a)] derived from an averaged MRI^21^ was used with optical coefficients from Cassano et al.^18^ (at 810 nm) displayed in Table 1.

**Table 1:** Optical properties used in this study for the layers of the simulated volume mesh. The values are taken from Cassano et al.^18^ at 810 nm.

	$\mu_{a}$ (${\mathrm{mm}}^{-1}$)	$\mu_{s}$ (${\mathrm{mm}}^{-1}$)	g	n
Skin	0.045	19.818	0.89	1.37
Skull	0.011	17.4545	0.89	1.37
CSF	0.0026	0.0909	0.89	1.37
GM	0.028	7.3	0.89	1.37
WM	0.092	38	0.87	1.37
Air cavities	0	0	1	1

A uniformly distributed collimated disk source with 2 in. diameter was directed toward the side of the head ∼4  cm above the tip of the ear.

Figure 1(b) shows the attenuation of fluence inside the head from 1 cm to 15.5 cm depth is of order $10^{16}$ to $10^{18}$. The graph in Fig. 1(b) represents the sum of the fluence for voxels inside the head in sagittal slices that are increasingly further from the source. The values were normalized by the slice with the maximum total fluence. The increase in normalized fluence for slices up to 1 cm depth inside the head is due to the curvature of the head surface. Only voxels inside the head are used in the summation, and some areas of the source do not interact with the tissue until deeper slices. Section 1 in the Supplementary Material explains this in more detail with accompanying diagrams.

To overcome such high attenuation factors and adequately sample the time-of-flight (ToF) distribution at the detector, 362 simulations were performed across three computers with NVIDIA GeForce RTX 4090 graphics cards for a combined run time of >850  h. The graphics card memory could run $2\times 10^{11}$ single-point precision photon packets per simulation, and the aggregated photon weights at the detector from a total of $7.24\times 10^{13}$ launched photons were used to estimate the final distribution.

Analysis of the simulated fluence-rate distribution through the head in Fig. 1(c) shows that light explores all regions of the brain and persists mostly in areas with low scattering and absorption, such as the cerebrospinal fluid above and below the cortex.

### 2.2 Experimental Methods

To demonstrate the feasibility of detecting photons that have experienced the longest trajectories, and therefore most likely to interact with the deepest layers of the head, an experiment was performed to detect light that has traveled diametrically from one side of the head to the other (i.e., across the widest point of the skull).

**Fig. 2:**
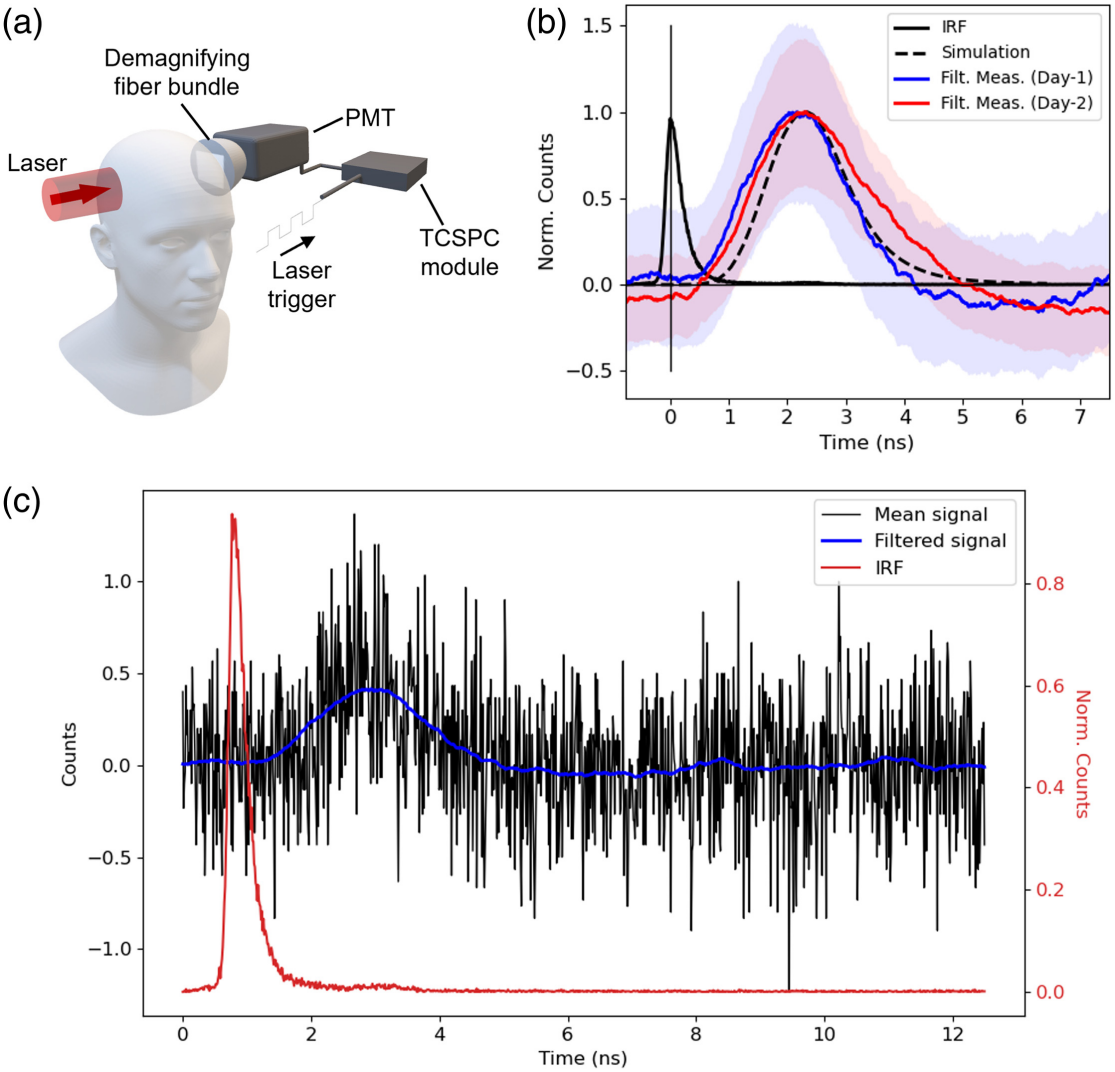
Experimental results. (a) The experimental configuration used in laboratory experiments—an ultrafast pulsed laser (1.2 W power, 800 nm wavelength, 140 fs pulse duration, 80 MHz repetition rate) is expanded to a uniformly distributed 1 in. diameter circle and projected against the side of the head. Diametrically opposite the source, a PMT is synchronized to the laser trigger such that a histogram of photon ToF can be measured using TCSPC. (b) The normalized simulated ToF (black dashed) and the mean of 15 experimental trials (2 min exposure) on day 1 (blue) and day 2 (red). The uncertainty bands indicate the standard error of the mean at each time stamp. The IRF (black) with the peak centered at time = 0 ns is shown for comparison. The start time of all time traces are aligned to the peak of the IRF. (c) The mean of the background-subtracted counts (black) of day 1 data in each timebin for 15 2-min exposures. The filtered mean counts for each timebin using a Savitzky–Golay filter (window = 251, order = 3). The normalized IRF is shown in red for comparison.

The experimental configuration is outlined in Fig. 2(a): a pulsed laser (1.2 W power, 800 nm wavelength, 140 fs pulse duration, 80 MHz repetition rate) is expanded to a uniformly distributed circular beam of 1 in. diameter and projected against the side of the head above the ear. Diametrically opposite the source, a demagnifying tapered fiber bundle (Edmund Optics, Barrington, New Jersey, United States, 25 to 8 mm fiber optic taper) is placed in close proximity to the scalp and redirects light to a photomultiplier tube (PMT, Hamamatsu, Japan, H7422P-50). The PMT operates in photon counting mode, such that the detection of a photon produces an electrical pulse that can be synchronized with the laser emission to produce a photon ToF distribution using a time-correlated single-photon counting (TCSPC) module (Becker & Hickl, Berlin, Germany, SPC-150N).

The combination of high laser power and a large-area single-photon sensitive detector is optimized to overcome the extreme attenuation of light through the head. Increasing the area of incident laser exposure allows the use of high powers without reaching the maximum permissible exposure of human skin. This is in contrast to typical fNIRS devices that use point-like emitters in an effort to maintain high spatial resolution, as well as low-power and small form-factor hardware.

The detector used in this work is chosen to maximize the etendue (product of light collection solid angle and sensor area) of light collection with a large-area detector (5 mm diameter). The demagnifying tapered fiber bundle is used to further increase the area of collection by ∼5× at the sacrifice of ∼5× reduction in solid angle of collection. Time-resolved DOT systems can be characterized using the basic instrument protocol outlined by Wabnitz et al.,^22^ which suggests that the maximum AΩ product of a typical DOT fiber collection system is limited to $∼8\;{\mathrm{mm}}^{2}\;\mathrm{sr}$. In our configuration, the PMT sensor is recessed by the outer casing limiting the angle of collection to an equivalent of NA=0.52. By the same calculation in the work by Wabnitz et al.,^22^ our configuration has $A\Omega =16.68\;{\mathrm{mm}}^{2}\;\mathrm{sr}$, more than twice the estimated maximum of typical systems. The PMT used in this work is commonly used in the field of fNIRS due to its high sensitivity to near-infrared wavelengths (QE = 15% at 800 nm) and the timing characteristics and sensitivity are reported by Wabnitz et al.^22^ We measure our system to have a 300 ps (full width half maximum) impulse response function (IRF) and a dark count rate of 15 cps. An advantage of time-resolving measurements in this experiment using TCSPC allowed us to further overcome background photon counts and noise that is uncorrelated with the laser source.

To prevent light from reaching the detector from sources other than light transmitted through the head, the experiment was performed in a light-tight enclosure that surrounded the head. The enclosure was built using black foamboard and covered with two layers of black cloth and a laser safety curtain. The participant’s torso from the waist upward was also wrapped in two layers of black cloth, which was sealed to the enclosure using Velcro. To prevent light scattering from optics immediately after the laser and reflecting from objects around the lab, a separate light-tight enclosure was built and similarly covered with cloth and the laser safety curtain. A black silicone mold with a 1 in. inner diameter hole was used to press the source against the head and prevent back reflections from the skin reaching the detector. On the detector side, sponge wrapped in black cloth was used to make a comfortable seal against the head during measurements and isolate the area of the head diametrically opposite the source from any other spurious reflections in the light-tight enclosure reaching the detector. Similarly, the detector was enclosed in a custom-built foamboard box wrapped with black cloth inside a larger foamboard enclosure, which was also covered with cloth and the laser safety curtain. Light reaching the detector from reflections around the lab appeared as a time-delayed impulse response function with 300 ps full width half maximum. Improvements in the seals around the experiment that extinguished this signal were used to ensure the enclosure was light-tight. A picture of the enclosures can be found in the Supplementary Material.

## 3 Results

### 3.1 Experimental Photon Detection

The results in Fig. 2(b) show experimental ToF distributions for photons transmitted through an adult male head (15.5 cm diameter) with fair skin and no head hair, collected on two different days, 1 week apart. For each day, the time traces are the mean of 15 2-min exposures, resulting in 30 min of total acquisition time. In every five exposures, a background reading was recorded where the subject was in the same position, but the laser beam shutter was activated to prevent light from reaching the head. The average counts in the background readings were subtracted from the average counts in the signal recordings for each timebin, and the result was smoothed using a Savitzky–Golay filter (window = 251, order = 3). An example of the mean background–subtracted photon counts overlayed with the filtered signal is shown in Fig. 2(c). The standard error shown in Fig. 2(b) for each timebin was calculated using the 2 min exposures before filtering. The standard error was added to and subtracted from the filtered signal to estimate the upper and lower bounds of the counts in each timebin, respectively. The upper and lower bounds were smoothed with the same filter used for the signal. For detailed inspection, Fig. 2(b) is plotted over a log scale in Sec. 2 of the Supplementary Material.

The measured experimental attenuation was found to be of the order $10^{18}$, corresponding to a detection of around one photon per second for a 1.2 W source. The simulated ToF distribution for the experimental configuration was approximated using a 2 in. diameter uniformly distributed source and a detector area corresponding to the photons leaving the surface of the head mesh cropped to a depth 13.3 cm from the source onward, above the ear, and behind the temple. The source size is larger than in the experiments to account for the challenging repeatability of source placements between measurements. The underlying population distribution was approximated using kernel density estimation using kernel bandwidths determined by Silverman’s rule^23^ to overcome subsampling. The distribution is then convolved with the experimental impulse response function. The simulated result shows good agreement with experimental results for the first two moments of the distribution, i.e., for both the peak delay time and width of the ToF distribution, indicating that the measured signal propagated through the head via similar migration pathways as seen in the numerical model. We attribute the discrepancy between the tails of the experimental distributions on different days to the challenging reproducibility of the source and detector placements between trials and the low number of photons measured, which results in a low signal-to-noise ratio. Despite this, we still see the strong overlap of the uncertainty bounds represented by the standard error on the mean photon counts in each timebin. Further analysis of the experimental data with the simulated time-resolved fluence data for increasing depths is shown in Sec. 3 of the Supplementary Material.

### 3.2 Photon Migration Pathways

Despite considering photon propagation in the human head as a diffuse optics problem where it is typically expected that light is highly scattered in all directions, the heterogeneity of optical properties and complex geometry of different layers causes light to be guided through the head in preferred pathways. This phenomenon is caused by channels of low scattering and absorption (e.g., cerebrospinal fluid) surrounded by high scattering (e.g., skull and gray matter), such that light follows the path of least extinction. A general study of the fundamental mechanism of guided light in diffusive materials was recently explored by Mitchell et al.^24^ In the context of fNIRS, light guided by cerebrospinal fluid has been observed in numerous previous studies and is typically considered an unwanted nuisance that decreases the depth of penetration of light and increases uncertainty when trying to locate brain activity.^25^^–^^27^ By contrast, in this work, we argue that the role of light guided by weakly scattering channels is critical for localizing changes in light absorption in deep brain regions and can be exploited by optimized source and detector arrangements.

**Fig. 3:**
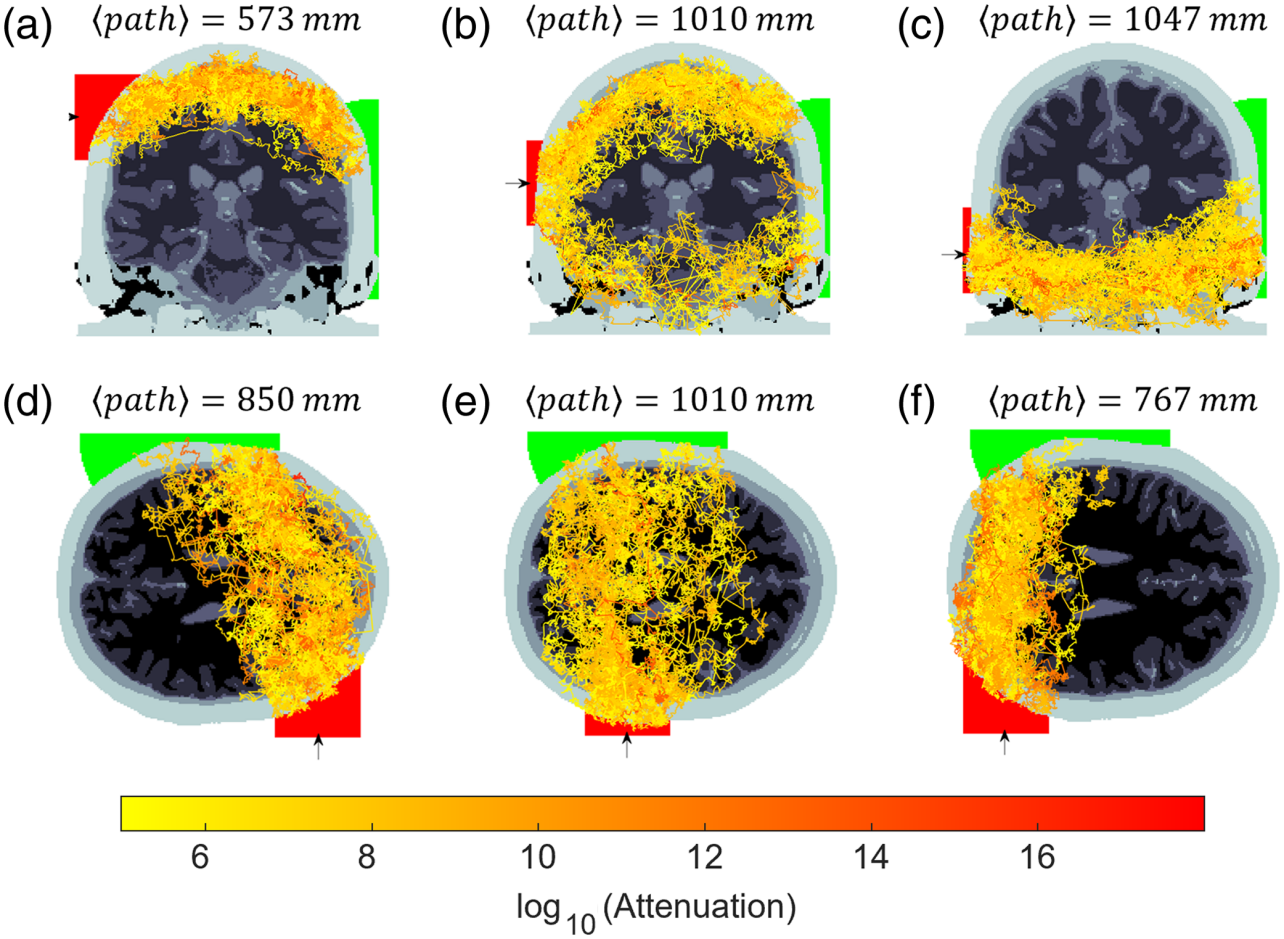
Numerical simulations: a two-dimensional projection of 50 random transmitted photon paths when moving the source, represented by an arrow and red area (2 in. diameter) outside of the head from the top to the bottom [(a)–(c)] and from the front to the back of the head [(d)–(f)]. The positions in panels (b) and (e) are approximately the positions of the source and detector in experimental measurements. The expectation value of the photon packet path length is displayed above each image. The detector area is the intersection of a sphere (green) and the surface of the head, which remains fixed in all cases.

Analysis of 50 random detected photon packet trajectories for various source positions is shown in Fig. 3. Positioning the source high above the ear can cause light to be guided around the top of the brain in the CSF layer [Fig. 3(a)], whereas lower positions cause light to be transported under the brain [Fig. 3(c)]. Likewise, moving the source toward the back of the head or forward toward the temple can increase the likelihood of photon pathways reaching the occipital or frontal lobe regions, respectively [Figs. 3(d)–3(e)].

The position of the source in Figs. 3(b) and 3(e) closely approximates the configuration used in the experiment. A source launch area of 2 in. diameter was used to account for the uncertainty in position throughout the experiments. A detector with a radius of 4 cm was placed diametrically opposite the source and remained fixed for all source positions. Surprisingly, although it is likely that most of the experimentally detected photons propagated around the top of the head, Fig. 3(b) indicates that there are also trajectories under the cerebrum with similar order of magnitude attenuation.

The expectation value of the photon packet path lengths ⟨path⟩ for the configuration in Figs. 3(b) and 3(e) is over 1 m propagation length. Normalizing the photon packet path lengths by the source-detector separation SDD=155  mm corresponds to a differential path length factor of $\mathrm{DPF}=\frac{\left\langle \text{path}\right\rangle }{\mathrm{SDD}}=6.5$, which matches the measured and predicted values from conventional DOT systems for an adult human head.^28^ Of the total DPF, the partial path length contributions for each layer are as follows: 17% from scalp, 35% from skull, 22% from cerebrospinal fluid, 19% from gray matter, 6% from white matter, and 1% from air cavities. Okada et al.^29^ report typical partial path contributions for DOT with SDD = 50 mm to be: 65% from scalp and skull, 35% from the cerebrospinal fluid, and 5% in the gray matter. If the signal-to-noise ratio could be improved, a possible advantage of transmission geometry measurements is the increased proportion of the photon path interacting with gray matter compared with reflection geometries, which could lead to greater sensitivity to cerebral hemodynamics.

**Fig. 4:**
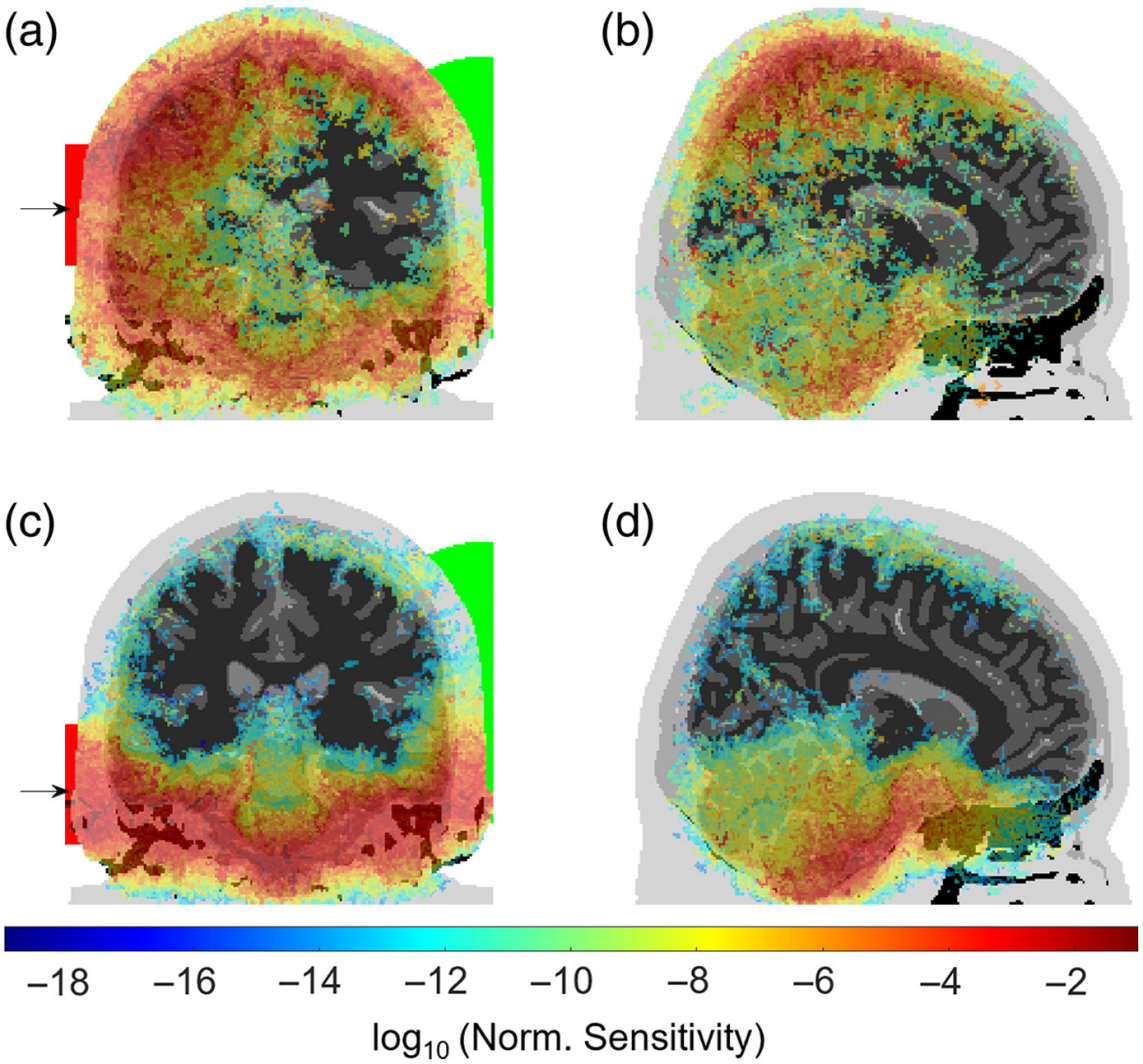
The sensitivity map (Jacobian matrix) for the source position approximately matching the experimental conditions [(a) and (b)] and 40 mm lower than experimental conditions [(c) and (d)]. The detector area is the intersection of a sphere (green) and the surface of the head.

Unlike conventional systems, the large source-detector separation results in a much greater number of total transport mean free path lengths experienced by a photon. The weights of the detected photon packets $w_{i}$ in the i’th layer were used to calculate a weighted average of the reduced scattering $\langle \mu_{s}^{\prime }\rangle =\overset{N}{\underset{i}{\sum }}w_{i}\mu_{s_{i}}^{\prime }/\overset{N}{\underset{i}{\sum }}w_{i}$ and the absorption coefficient $\left\langle \mu_{a}\right\rangle$ for N layers described in Table 1. The expected number of transport mean free paths experienced by a photon from the source to the detector is therefore $\mathrm{SDD}\times (\langle \mu_{s}^{\prime }\rangle +\langle \mu_{a}\rangle )=234$. Using typical values used in reflection geometries ($\langle \mu_{s}^{\prime }\rangle =1\;{\mathrm{mm}}^{-1}$, $\langle \mu_{a}\rangle =0.1\;{\mathrm{mm}}^{-1}$, SDD = 50 mm), we estimate most conventional DOT systems detect photons that have undergone ∼50 transport mean free path lengths. We also show in Fig. 4, a sensitivity analysis that is obtained by calculating the Jacobian for the light rays, i.e., a map of how light intensity changes at the detector for small changes to the absorption coefficient at each voxel in the head.^30^ The Jacobian for the source-detector arrangement approximating experimental conditions, Figs. 4(a) and 4(b), highlights that measurements in this configuration are indeed sensitive to changes in the absorption in deep regions of the brain reaching areas that are currently inaccessible for fNIRS devices, such as the midbrain, sulci, and deep regions of the cerebellum. Figure 4(c) shows that by repositioning the source 40 mm lower, the sensitivity map is isolated almost exclusively to regions under the cerebrum and demonstrates the potential to target deep brain regions with careful consideration of source-detector arrangements. These Jacobians are an extension of the typical “banana-like” sensitivity profiles found in fNIRS^1^^,^^31^ in the limit of large source-detector separation, which here evolve into nontrivial shapes that could be used to reconstruct tomographic information.

## 4 Discussion and Conclusion

Detecting photons transported through large and diametrically opposite source-detector separations has the potential to extend the field of optical brain imaging devices to reach regions of the brain currently considered inaccessible. Experimental measurements of photons transmitted through the entire adult human head suggest that, although the attenuation of light is challenging, in principle, it is possible to detect light from the most extreme source-detector separations. We speculate that the participant’s fair skin and lack of hair were significant factors that reduced the attenuation of light to feasibly detect a signal. In addition to the participant wherein a signal was observed, the experiment also included trials on seven other subjects. The details of the subject pool are as follows: two females and six males; 25 to 35 years old; 14.5 to 15.5 cm head diameter; Fitzpatrick skin types: 3 type I, 4 type II, and 1 type V; hair types: 1 bald, 4 short and light-colored, and three dense and dark-colored. We did not observe any significant time-correlated signals above background noise for the seven other subjects.

We also acknowledge that 30 min to acquire a reasonable signal is unfeasible for most practical applications e.g., monitoring cerebral hemodynamics or rapid point-of-care diagnostics. However, the focus of this work is to show that it is possible to detect light transmitted in the most extreme source-detector configuration; that light is not back-reflected from shallow regions but has migrated through the entirety of the head. Configurations that are between typical fNIRS reflection paradigms and the worst-case transmission regime presented in this work would have a reduced number of attenuation lengths and therefore an exponential increase in signal that would likely be sufficient for some practical applications. Furthermore, the work presented here may have a greater impact on static imaging applications that do not require fast sample rates, such as detecting brain hemorrhage, traumatic brain injury, or tumors.

Related works that detect time-resolved signal changes due to changes in the absorption in the brain at large source-detector separation up to 9 cm were reported by Liebert et al.^32^^,^^33^ These works report similar conclusions for extending the conventional DOT paradigms to detect deep brain activity, namely that a high-power source incident over a large incidence area is required and the collection efficiency should be improved by replacing collection fibers with high NA, large-area sensors in close proximity to the skin. Liebert et al.^33^ report a maximum depth of sensitivity to absorption changes of 5 cm with a practical sample rate of 10 Hz. This supports the idea that the higher power and larger collection area regime presented in our work placed at source-detector separations greater than 9 cm but less than the diameter of the head could lead to practical signal-to-noise ratios to perform deep hemodynamic activity monitoring.

Although we find that the numerical simulation of the experimental measurement has a relatively close qualitative agreement with the experimental data, we expect discrepancies given the large uncertainty of optical properties of the head layers *in vivo* which can vary by 100% in the literature.^34^^,^^35^ We also expect deviations between the simulated and experimental data due to the differences in structure, shape, and thickness of the layers between the open-source mesh used in simulations versus the participant’s anatomy. Although this will almost certainly cause the simulated photon migration pathways to differ from reality in the fine details, we expect the observed guiding principle, e.g., above and below the cerebrum to remain accurate.

Furthermore, careful consideration of source-detector configuration and ToF analysis present an opportunity to selectively isolate photons that are confined to guided propagation pathways that could possibly be combined to reconstruct tomographic information of deep brain activity. Due to the necessary requirement of large source and detector areas to detect photons transmitted through large distances across the head, combined with the extent of inherent scattering over these distances, the Jacobian covers a much greater volume than those used in conventional DOT imaging systems. Although we do not propose the details of an inverse image reconstruction algorithm, we do believe that combining measurements for different source-detector configurations could be used in future studies to localize absorption in targeted brain regions with optimal optode placement. We speculate that the large volume of the Jacobians for the regime used in this work suggests that image resolutions are unlikely to match the resolution of traditional systems that target the surface of the cortex.

Another interesting consideration is the wavelength dependency of the attenuation for such large length scales for longer wavelengths. Some studies^36^^–^^39^ have shown that short-wave infrared wavelengths have a lower reduced scattering and absorption for biological tissues that significantly improve signal-to-noise ratio. In this case, we expect that transmitting light through the head at wavelengths beyond 1  μm could make the physical problem of overcoming scattering and absorption less challenging. However, current single-photon detectors at short infrared wavelengths do not have the combination of a low dark count rate and a large solid angle of collection to detect the transmitted photons with the same efficiency as detectors in the near-infrared.

## Supplementary Material

10.1117/1.NPh.12.2.025014.s01

## Data Availability

Due to privacy concerns, supporting data cannot be made openly available. Further information about the data and conditions for access are available from the Enlighten: Research Data repository at 10.5525/gla.researchdata.1968.

## Declarations

Participants involved in this work provided informed consent before experiments, and the experiments were approved by the University of Glasgow College of Science and Engineering Ethics Committee (Application No. 300180292).
