# Supplementary material for: Photon transport through the entire adult human head
Source: Neurophotonics. 2025 May 28;12(2):025014. doi: 10.1117/1.NPh.12.2.025014 (PMC12117216; doi:10.1117/1.NPh.12.2.025014)
Supplement: Supplementary file 1 [file NPh_012_025014_SD001.pdf]

Supplementary material: Photon transport through the entire adult human head

Jack Radford[1], **Vytautas Gradauskas**[1], **Kevin J. Mitchell**[1], **Samuel Nerenberg**[1], **Ilya Starshynov**[1], **Daniele Faccio**[1*]
[1]School of Physics and Astronomy, University of Glasgow, Glasgow, G12 8QQ, UK

1 Fluence in shallow regions of the head

Plotting the normalised fluence inside the head for slices of increasing distance from the source was used to estimate the attenuation factor of light transmitted through the head in Figure 1b of the main text. However, the fluence appears to increase in shallow regions up to 1 cm. This is because the fluence calculated at each slice includes only the interactions with voxels inside the head and does not account for light that is reflected or has yet to reach the skin. Figure 1a) shows the area of the source in red that has yet to interact with the volume and is not included in the total fluence for the slices indicated with dashed lines. Figure 1b) shows the fluence values normalised by the maximum voxel in the entire head plotted in log-scale and the dashed circle represents the source position.

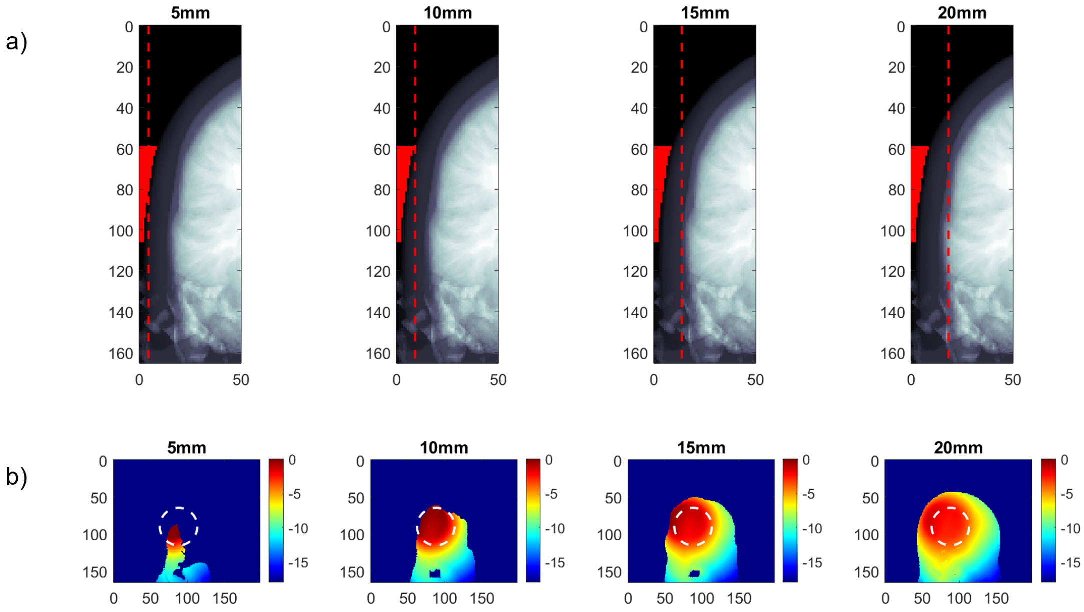

Fig 1 a) A coronal view of the head atlas used in simulations showing the regions of the incident source that is not included in the normalised fluence values. The dashed line represents the position of the slice into the head for b) the sagittal fluence distribution maps. For shallow slices, the beam has not yet interacted with the volume. The colourbar represents the normalised fluence rate in log-scale.

2 A comparison of measurements in log-scale

Figure 2 compares the normalised photon counts for the measurements, simulation and IRF using a log-scale. The IRF was measured in the absence of the subject's head, directing an attenuated beam toward a piece of paper placed in front of the detector. The small peak in the IRF at 3 ns is a characteristic of the PMT and matches the observations of a secondary peak occuring around 2.5 ns after the main peak reported in technical reports[17,22] for the same detector.

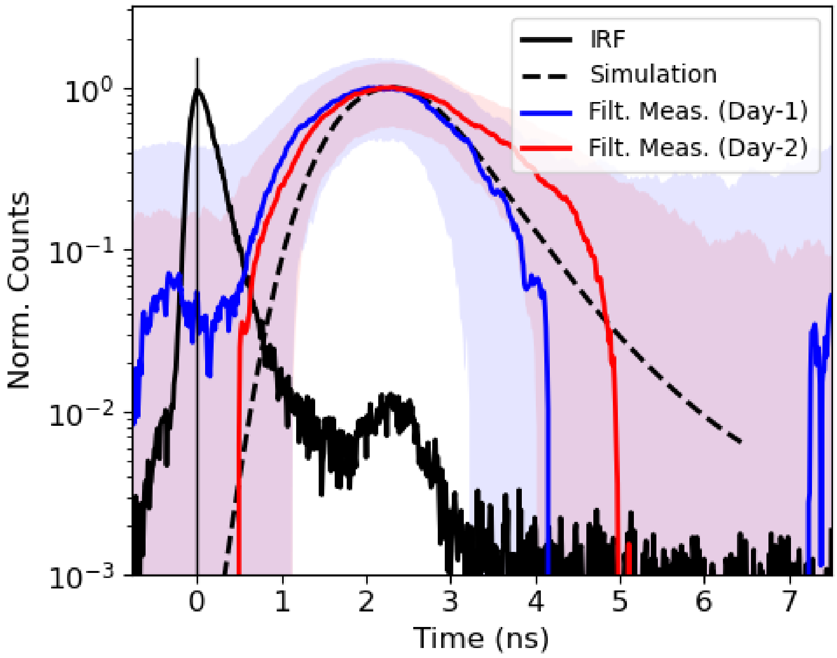

Fig 2 The normalised simulation, impulse response function (IRF), and filtered measurement data with standard error bounds plotted in log-scale.

3 Experimental validation with a phantom

To assess the validity of the experiment to measure time-resolved signals from diffusive materials, a polystyrene scattering phantom was used in place of a human head. The scattering phantom has stable optical properties and the homogeneity of the material makes the time-of-flight distribution at the detector easy to compare with a simple analytical model of diffuse light propagation. The experiment was identical to that described in Section 2.2 in the main manuscript, but a wide aperture lens was used (Meike MK-35mm, f/1.4) instead of a fiber taper, and in place of the subject's head, varying thicknesses of foam were used. Additional black cloth was used to pad the surrounding of the foam to prevent back reflections from reaching the detector.

Measurements were taken for increasing thicknesses of foam L by stacking $d = 2.58$ cm ± 0.01 cm slabs between the source and detector from $L = 5.16$ cm to $L = 15.48$ cm. The photons were collected at the detector for a total acquisition time of 120 seconds and the power of the source was adjusted for every measurement to avoid saturating the detector and maintain the photon-starved regime to avoid pile-up effects:

Thickness (cm)	Source Power (mW)
5.16	0.0261 ± 0.0001
7.74	1.25 ± 0.01
10.32	50.37 ± 0.04
12.90	402.36 ± 0.43
15.48	402.36 ± 0.43

We fit the absorption μ_a and reduced scattering μ_s' coefficients for each measurement using a least-squares optimisation to the time-resolved transmittance solution to the diffuse photon approximation with finite slab boundary conditions.[40] The simulations were time-shifted to compensate for the different impulse response function positions for slabs with increasing thickness.

Although the TCSPC card was set to trigger on every fourth pulse emitted by the laser, the laser repetition rate is fixed at 80 MHz which results in time-of-flight distributions from four laser pulses to be recorded in a 50 ns window. Time-of-flight curves for slabs with thickness $L > 7$ cm have widths greater than the laser repetition rate causing the last photons to arrive at the detector to overlap with the first photons to arrive from the subsequent pulse. The mean of two out of four recorded time-of-flight distributions which were not cropped by the regions at the start and end of the TCSPC window due to the non-linear regimes of the time-to-analogue converter, were used to fit the analytical equation. The analytical function was convolved with the impulse response function and the photons arriving later than 12.5 ns were wrapped and added to the start of the signal to match the experimental signal overlap and make use of all of the measured data points in the fitting process.

The results in figure 3b) show that for a controlled homogenous scattering phantom over different length scales, the analytical model fits closely with the time-of-flight distribution. Although the physical length scales are similar, these results cannot be compared to the measurements of diffusion transmitted through the head shown in the main text. The homogeneity of the scattering phantom will result in dramatically different results when compared to heterogeneous modeling of the human head. Attempting to approximate the head with a homogeneous scatterer and using average optical coefficients for the superficial layers of the head and grey matter ($\mu_a = 0.28\text{cm}^{-1}$, $\mu_s' = 14\text{cm}^{-1}$), would result in an attenuation of 10^{24}, corresponding to a detection rate of less than 1 photon per week for a 1W source. This significantly differs from the estimation of 10^{18} attenuation from Monte Carlo simulations and experimental data and underlines the significant contribution of light guided by the cerebrospinal layer for transmitting photons through the head. The results in figure 4b) show that as the depth of penetration in the head increases the light is predominantly localised in regions of cerebrospinal fluid and the time-of-flight converges to the peak position and width observed in experimental measurements shown in figure 2 of the main manuscript.

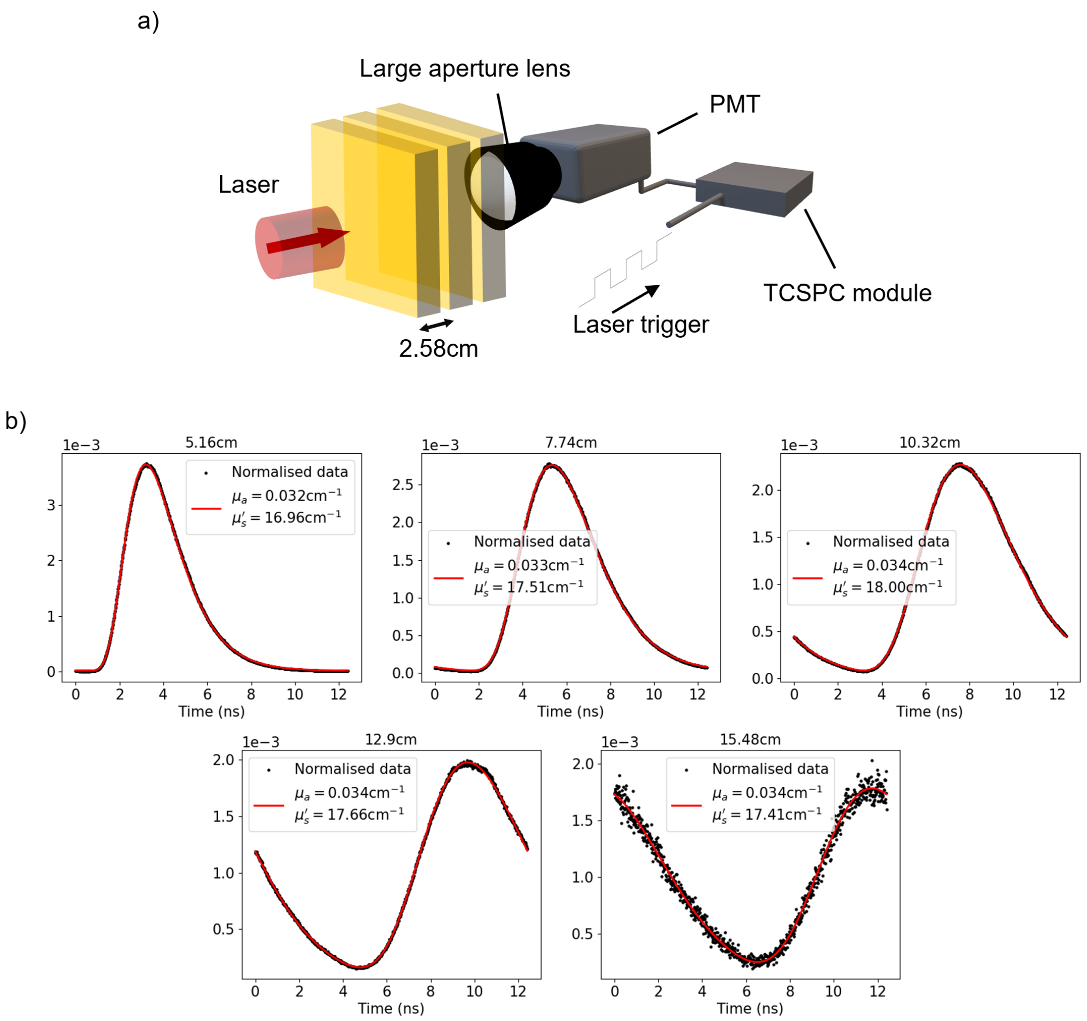

Fig 3 a) The experimental set up is identical to the one described in figure 2 of the main text where the head is replaced with polystyrene foam with similar average scattering properties to the human head and the fibre taper is replaced with a wide aperture lens. b) The experimental and analytical fits normalised by area for increasing thickness of foam.

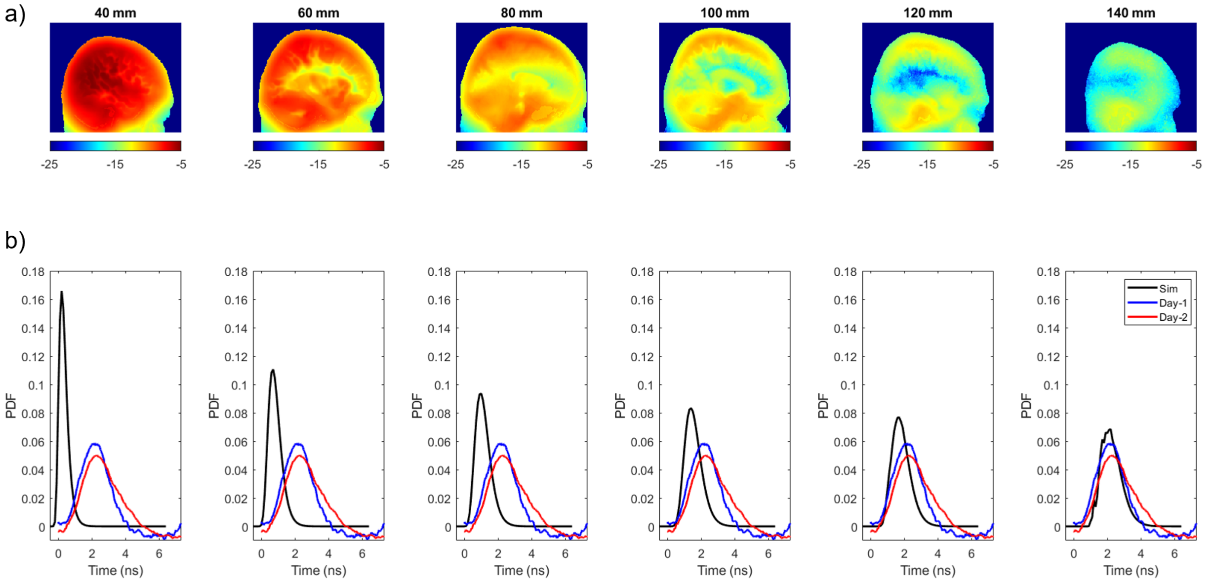

Fig 4 a) The normalised fluence in log-scale ($1/mm^2$) for sagittal slices in the simulations described in section 2.1 of the main manuscript. b) The probability density functions (PDF) for each of the slices shown in (a) (black) overlayed with the filtered day-1 (blue) and day-2 (red) experimental data.

4 Experiment enclosure

The experiments were performed in a light-tight enclosure made from black foamboard to prevent reflections from objects in the lab reaching the detector (Fig. 5). The source coupling optics and the detector were placed in separate enclosures neighbouring the one used for directing light to the head and all enclosures were covered with 2 layers of black cloth and a laser safety curtain.

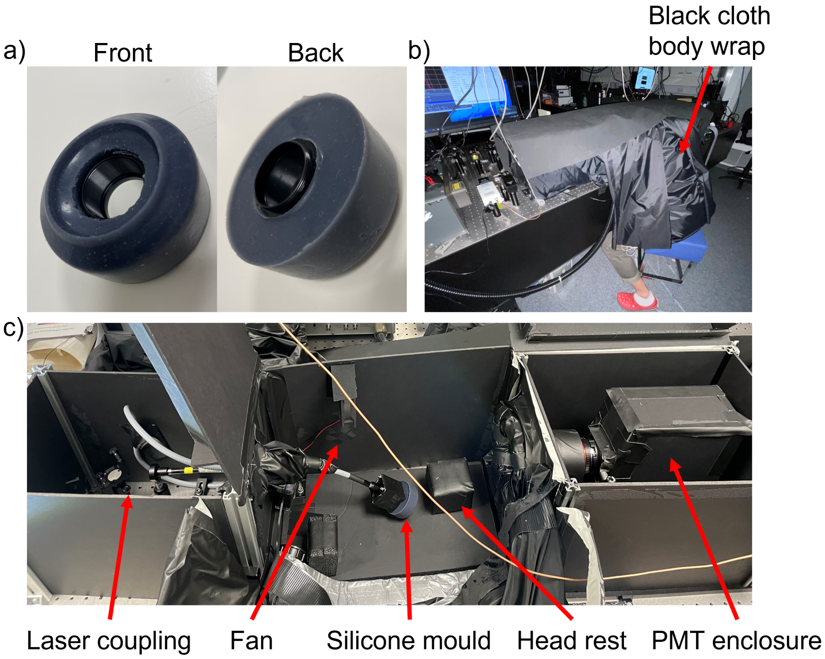

Fig 5 a) the silicone mould used to isolate the incident area of the source and prevent back reflections reaching the detector. b) A participant in the enclosure during a measurement. c) The enclosure with the lids removed. For the signals shown in the main text the lens shown in this image was replaced with a fiber optic taper.

A silicone mould made using the bottom of an aluminium drinks can with a 1-inch inner diameter hole was used to make a seal around the area of laser incidence and prevent back reflection from the skin reaching the detector. Diametrically opposite the source, sponge wrapped in black cloth was used to make a seal against the head and prevent light from reflections inside the enclosure reaching the detector. The torso of the participant from the waist upwards was wrapped in black cloth which was attached to the enclosure using velcro.
